# Supplementary material for: QM/MM Modeling of the Flavin Functionalization in the RutA Monooxygenase
Source: Molecules. 2023 Mar 6;28(5):2405. doi: 10.3390/molecules28052405 (PMC10005588; doi:10.3390/molecules28052405)
Supplement: Supplementary file 1 [file molecules-28-02405-s001.zip › molecules-2213118-supplementary.pdf]

Supporting Materials

for

QM/MM modeling of the flavin functionalization in the RutA monooxygenase

Bella L. Grigorenko [1,2], Tatiana M. Domratcheva [1] and Alexander V. Nemukhin [1,2,*]

[1] Department of Chemistry, M.V. Lomonosov Moscow State University, Moscow, 119991, Russia

[2] N.M. Emanuel Institute of Biochemical Physics, Russian Academy of Sciences, Moscow, 119334, Russia

* Corresponding author:

Prof. Alexander V. Nemukhin, Chemistry Department, Lomonosov Moscow State University, Leninskie Gory 1/3, Moscow, 119991, Russian Federation

E-mail: anem@lcc.chem.msu.ru

Section S1.

Description of the files with the atomic coordinates (pdb-format) of the structures optimized in QM/MM calculations, which are deposited to the general-purpose open-access repository ZENODO (can be accessed via https://doi.org/10.5281/zenodo.7558382).

Complex-1.pdb - the minimum-energy structure of the triplet-state flavin-oxygen complex, which initiates pathway-1 (Fig. 2a in the manuscript).

Complex-2.pdb - the minimum-energy structure of the triplet-state flavin-oxygen complex, which initiates pathway-2 (Fig. 4a in the manuscript).

Complex-3.pdb - the minimum-energy structure of the triplet-state flavin-oxygen complex, which initiates pathway-3 (Fig. 5a in the manuscript).

Complex-4.pdb - the minimum-energy structure of the triplet-state flavin-oxygen complex, which initiates pathway-4 (Fig. 7a in the manuscript).

Complex-5.pdb - the minimum-energy structure of the triplet-state flavin-oxygen complex, which initiates pathway-5 (Fig. 9a in the manuscript).

1-FlC4a.pdb – the minimum-energy structure of the singlet-state flavin-oxygen adduct Fl_{C4aOO}^{-} located on the pathway-1 (Fig. 2b in the manuscript).

2-Flox.pdb – the singlet-state minimum-energy structure of the oxidized flavin with the nearby protonated superoxide located on the pathway-2 (Fig. 4b in the manuscript).

3-Flox.pdb – the singlet-state minimum-energy structure of the oxidized flavin with the nearby hydrogen peroxide and hydroxyl located on the pathway-3 (Fig. 5c in the manuscript).

4-FlN5O.pdb – the minimum-energy structure of the singlet-state flavin-oxygen adduct Fl_{N5O} located on the pathway-4 (Fig. 8a in the manuscript).

4-FlN5O-C8.pdb – the minimum-energy structure of the singlet-state flavin-oxygen adduct Fl_{N5O} hydrated at the C8 position, which is located on the pathway-4 (Fig. 8b in the manuscript).

5-FlC6.pdb – the minimum-energy structure of the singlet-state flavin-oxygen adduct Fl_{C6OOH} located on the pathway-5 (Fig. 9b in the manuscript).

5-FlC6C7.pdb – the minimum-energy structure of the singlet-state flavin-oxygen adduct with the C6-C7-epoxide located on the pathway-5 (Fig. 9c in the manuscript).

Section S2.

A sample input file for the QM/MM optimization using the NWChem program

```
title "fl4sb"
start fl4sb
echo

charge -4
memory total 3000 mb
#print debug
SCRATCH_DIR /tmp
PERMANENT_DIR .

prepare
    directory_3 ../
    system fl4sb
    source ./fl4sb-F.pdb
    amber
    new_top new_seq
    new_rst
    modify atom    1:_N1   quantum
    modify atom    1:_H1   quantum
    modify atom    1:_C2   quantum
    modify atom    1:_O2   quantum
    modify atom    1:_N3   quantum
    modify atom    1:_H3   quantum
    modify atom    1:_C4   quantum
    modify atom    1:_O4   quantum
    modify atom    1:_C4A  quantum
    modify atom    1:_N5   quantum
    modify atom    1:_C5A  quantum
    modify atom    1:_C6   quantum
    modify atom    1:_H6   quantum
    modify atom    1:_C7   quantum
    modify atom    1:_C7M  quantum
    modify atom    1:_H71  quantum
    modify atom    1:_H72  quantum
    modify atom    1:_H73  quantum
    modify atom    1:_C8   quantum
    modify atom    1:_C8M  quantum
    modify atom    1:_H81  quantum
```

```
modify atom    1:_H82  quantum
modify atom    1:_H83  quantum
modify atom    1:_C9   quantum
modify atom    1:_H9   quantum
modify atom    1:_C9A  quantum
modify atom    1:_N10  quantum
modify atom    1:_C10  quantum
modify atom    1:_C1*  quantum
modify atom    1:2H1*  quantum
modify atom    1:3H1*  quantum
modify atom    1:_C2*  quantum
modify atom    1:_H2*  quantum
modify atom    1:_O2*  quantum
modify atom    1:2HO*  quantum
modify atom    1:_C3*  quantum
modify atom    1:_H3*  quantum
modify atom    1:_O3*  quantum
modify atom    1:3HO*  quantum
modify atom    1:_C4*  quantum
modify atom    1:_H4*  quantum
modify atom    1:_O4*  quantum
modify atom    1:4HO*  quantum
modify atom    1:_C5*  quantum
modify atom    1:2H5*  quantum
modify atom    1:3H5*  quantum
modify atom    1:_O5*  quantum
modify atom    1:_P    quantum
modify atom    1:_O1P  quantum
modify atom    1:_O2P  quantum
modify atom    1:_O3P  quantum
modify atom  105:_CB   quantum
modify atom  105:_HB   quantum
modify atom  105:_CG2  quantum
modify atom  105:2HG2  quantum
modify atom  105:3HG2  quantum
modify atom  105:4HG2  quantum
modify atom  105:_OG1  quantum
modify atom  105:_HG1  quantum
modify atom  134:_CB   quantum
modify atom  134:2HB   quantum
modify atom  134:3HB   quantum
modify atom  134:_CG   quantum
modify atom  134:_OD1  quantum
modify atom  134:_ND2  quantum
modify atom  134:2HD2  quantum
modify atom  134:3HD2  quantum
modify atom  139:_CB   quantum
modify atom  139:2HB   quantum
modify atom  139:3HB   quantum
modify atom  139:_CG   quantum
modify atom  139:_CD1  quantum
modify atom  139:_HD1  quantum
modify atom  139:_NE1  quantum
modify atom  139:_HE1  quantum
modify atom  139:_CE2  quantum
modify atom  139:_CZ2  quantum
modify atom  139:_HZ2  quantum
modify atom  139:_CH2  quantum
modify atom  139:_HH2  quantum
modify atom  139:_CZ3  quantum
```

```
modify atom  139:_HZ3  quantum
modify atom  139:_CE3  quantum
modify atom  139:_HE3  quantum
modify atom  139:_CD2  quantum
modify atom  292:_CG   quantum
modify atom  292:2HG   quantum
modify atom  292:3HG   quantum
modify atom  292:_CD   quantum
modify atom  292:_OE1  quantum
modify atom  292:_OE2  quantum
modify atom  400:_OX1  quantum
modify atom  400:_OX2  quantum
modify atom  501:2HW   quantum
modify atom  501:_OW   quantum
modify atom  501:3HW   quantum
modify atom  502:2HW   quantum
modify atom  502:_OW   quantum
modify atom  502:3HW   quantum
modify atom  503:2HW   quantum
modify atom  503:_OW   quantum
modify atom  503:3HW   quantum
modify atom  504:2HW   quantum
modify atom  504:_OW   quantum
modify atom  504:3HW   quantum
modify atom  505:2HW   quantum
modify atom  505:_OW   quantum
modify atom  505:3HW   quantum
modify atom  506:2HW   quantum
modify atom  506:_OW   quantum
modify atom  506:3HW   quantum
modify atom  507:2HW   quantum
modify atom  507:_OW   quantum
modify atom  507:3HW   quantum
modify atom  508:2HW   quantum
modify atom  508:_OW   quantum
modify atom  508:3HW   quantum
modify atom  509:2HW   quantum
modify atom  509:_OW   quantum
modify atom  509:3HW   quantum
modify atom  510:2HW   quantum
modify atom  510:_OW   quantum
modify atom  510:3HW   quantum
modify atom  511:2HW   quantum
modify atom  511:_OW   quantum
modify atom  511:3HW   quantum
modify atom  512:2HW   quantum
modify atom  512:_OW   quantum
modify atom  512:3HW   quantum
modify atom  513:2HW   quantum
modify atom  513:_OW   quantum
modify atom  513:3HW   quantum
modify atom  628:2HW   quantum
modify atom  628:_OW   quantum
modify atom  628:3HW   quantum
modify atom 2811:2HW   quantum
modify atom 2811:_OW   quantum
modify atom 2811:3HW   quantum
modify atom  514:2HW   quantum
modify atom  514:_OW   quantum
modify atom  514:3HW   quantum
```

```
    modify atom  600:2HW   quantum
    modify atom  600:_OW   quantum
    modify atom  600:3HW   quantum
    modify atom  619:2HW   quantum
    modify atom  619:_OW   quantum
    modify atom  619:3HW   quantum
    modify atom 2824:2HW   quantum
    modify atom 2824:_OW   quantum
    modify atom 2824:3HW   quantum
    update lists
    ignore
    write fl4sb.rst
    write fl4sb.pdb
end

task prepare

driver
    tight
end

md
    system fl4sb
    noshake solute
    cutoff 10 qmmm 10
    msa 30000
end

basis
#    * library "cc-pvdz"
    * library "6-31G*"
end

dft
    xc pbe0
    disp vdw 3
    iterations 250
    mult 1
    grid
end


driver
    trust 0.05
    tight
end

qmmm
    region qmlink mm_solute
    method bfgs lbfgs
    maxiter  5  100
    density espfit
    ncycles 100
    convergence 1.0d-7
    bqzone 50
end

constraints
#    spring bond  100   101   1.0   3.5
end
```

```
task qmmm dft optimize


prepare
    system fl4sb
    read rst fl4sb.rst
    write solute pdb fl4sb-F.pdb
end

task prepare
```

Section S3.

A sample input file for CASSCF and XMCQDPT2 calculations using the Firefly program.

```
!
 $intgrl extfi=.t. $end
 $MCQGENS GEN1=3 GEN2=3 GEN3=1 USEGEN1=.T. $END
 $xmcqdpt  halloc=.t. ri=.t.  alttrf(1)=1,1,1,1 bigcas=1 $end
 $rimp2 auxbas=def2-TZVPPD/C extfil=.t. $end
 $gugem pack2=1 $end
 $p2p p2p=1 dlb=1 $end
 $smp smppar=1 np=10 mklnp=10 $end
 $trans dirtrf=.t. aoints=dist altpar=.t. mptran=2 mode=112 $end
!
$CONTRL SCFTYP=mcscf RUNTYP=energy INTTYP=HONDO ICUT=10
    icharg=-1 mult=1
    maxit=350
!     dfttyp=pbe0
    mplevl=2
!     nzvar=1
 $END
 $ZMAT DLC=.T. AUTO=.T.
   NONVDW(1)= 10,48,  48,60,  48,77,  77,68,  77,75,
            75,51,  51,42,  42,45,  45,62,  62,65,
            56,62,  34,53,  34,72
!              ifzmat(1)=1,7,77
 $END
 $system TIMLIM=90000 mwords=300 $end
!
! Note, high computation accuracy is enforced throughout
!
 $trans dirtrf=.t. mptran=2 mode=0 cuttrf=1.0d-10 $end
 $statpt nstep=1000 opttol=1.d-4 projct=.f.
!      dxmax=0.02 trmax=0.02
!       ifreez(1)=1,2,3,  118,119,120,  130,131,132,
!              262,263,264,  178,179,180,  235,236,237,55, 56,57
$END
```

```
 $SCF DIIS=.t. SOSCF=.f. DIRSCF=.T.  $END
 $BASIS gbasis=cc-pvdz extfil=.t. $END
! $BASIS GBASIS=N31 NGAUSS=6 ndfunc=1 $END
! $dft nrad0=96 nleb0=302 $end
 $DRT GROUP=C1 Nmcc=170 NDOC=7 NALP=0 NVAL=3 FORS=.T. $END
 $GUGDIA NSTATE=20 ITERMX=3200 $END
 $gugdm2 wstate(1)=1,1,1,1,1, 1,1,1,1,1, 1,1,1 $end
 $MCSCF cistep=guga
!     istate=2 acurcy=1.0d-7 npflg(9)=1
      SOSCF=.T. FULLNR=.F. FCORE=.F. maxit=20
 $END
 $xmcqdpt istsym=1 kstate(1)=1,1,1,1,1, 1,1,1,1,1, 1,1,1,1,1
    irot=2 edshft=0.02 avecoe(1)=1,1,1,1,1, 1,1,1,1,1, 1,1,1,1,1
    inorb=0
 $end
 $GUESS  GUESS=MOREAD NORB=180 norder=0
   iorder(173)=170       iorder(170)=173
 $END
 $NBO reson aonbo=w $END
 $license nbolid=1 nbokey=57c9e756 $end
 $DATA

C1
 N    7.0          96.174  77.625  -3.754
 H    1.0          92.342  80.461  -2.037
 C    6.0          95.628  77.798  -4.973
 O    8.0          96.262  77.507  -6.004
 N    7.0          94.333  78.282  -5.105
 H    1.0          93.882  78.117  -6.008
 C    6.0          93.589  78.826  -4.089
 O    8.0          92.463  79.270  -4.239
 C    6.0          94.297  78.820  -2.786
 N    7.0          93.733  79.406  -1.765
 C    6.0          94.270  79.251  -0.536
 C    6.0          93.585  79.752   0.585
 H    1.0          92.670  80.316   0.437
 C    6.0          94.013  79.482   1.864
 C    6.0          93.120  79.869   3.006
 H    1.0          93.607  79.779   3.978
 H    1.0          92.247  79.206   3.016
 H    1.0          92.725  80.880   2.873
 C    6.0          95.254  78.802   2.044
 C    6.0          95.822  78.585   3.415
 H    1.0          96.818  78.141   3.362
 H    1.0          95.180  77.916   4.000
 H    1.0          95.898  79.530   3.964
 C    6.0          95.972  78.349   0.948
```

H	1.0	96.936	77.884	1.120
C	6.0	95.465	78.504	-0.348
N	7.0	96.076	77.974	-1.462
C	6.0	95.535	78.108	-2.697
C	6.0	97.392	77.366	-1.328
H	1.0	97.389	76.706	-0.460
H	1.0	97.572	76.757	-2.208
C	6.0	98.433	78.480	-1.231
H	1.0	98.228	79.085	-0.345
O	8.0	98.311	79.359	-2.329
H	1.0	98.725	78.946	-3.125
C	6.0	99.835	77.914	-1.082
H	1.0	100.522	78.764	-1.140
O	8.0	100.211	77.068	-2.157
H	1.0	99.788	76.186	-2.051
HL	1.0	99.986	77.410	-0.127
O	8.0	91.491	80.953	-1.904
O	8.0	90.719	80.031	-1.129
H	1.0	93.955	76.439	-8.369
O	8.0	93.780	77.277	-7.884
H	1.0	92.826	77.466	-8.006
H	1.0	98.382	74.329	-1.204
O	8.0	99.234	74.520	-1.676
H	1.0	99.157	74.042	-2.515
H	1.0	91.174	76.774	-0.885
O	8.0	90.538	75.366	-0.382
H	1.0	91.088	75.005	0.353
H	1.0	91.844	76.767	-4.870
O	8.0	91.772	76.703	-5.838
H	1.0	92.425	76.005	-6.021
H	1.0	93.875	72.083	-3.383
O	8.0	93.241	72.536	-4.000
H	1.0	92.578	73.035	-3.441
H	1.0	93.394	75.735	-4.085
O	8.0	92.923	76.086	-3.295
H	1.0	92.263	75.291	-3.060
H	1.0	94.033	74.432	-1.108
O	8.0	94.590	75.239	-1.331
H	1.0	94.103	75.634	-2.097
H	1.0	94.024	76.059	0.146
O	8.0	93.466	76.474	0.843
H	1.0	92.838	76.989	0.298
H	1.0	96.438	73.574	0.133
O	8.0	96.868	74.145	-0.537
H	1.0	96.114	74.592	-0.991
H	1.0	93.457	72.384	-0.800

O	8.0	92.997	73.270	-0.705
H	1.0	92.317	73.404	-1.435
H	1.0	93.727	73.823	-4.861
O	8.0	93.741	74.617	-5.476
H	1.0	94.644	74.673	-5.869
H	1.0	92.490	73.762	0.798
O	8.0	92.153	74.254	1.592
H	1.0	92.670	75.098	1.530
H	1.0	92.186	77.220	-1.997
O	8.0	91.768	77.534	-1.172
H	1.0	91.130	79.132	-1.284
H	1.0	90.806	74.833	-1.172
O	8.0	91.577	74.117	-2.622
H	1.0	90.696	73.923	-2.970
H	1.0	94.955	78.635	-8.245
O	8.0	95.812	79.114	-8.237
H	1.0	96.230	78.675	-7.475
H	1.0	95.932	75.999	-6.713
O	8.0	95.911	75.051	-6.971
H	1.0	95.275	74.985	-7.747
H	1.0	90.789	81.054	0.429
O	8.0	91.142	81.711	1.057
H	1.0	91.503	82.396	0.476

$END
